# Cardiotoxicity Associated with Anti-CD19 Chimeric Antigen Receptor T-Cell (CAR-T) Therapy: Recognition, Risk Factors, and Management

**DOI:** 10.3390/diseases9010020

**Published:** 2021-03-17

**Authors:** Ethan A. Burns, Cesar Gentille, Barry Trachtenberg, Sai Ravi Pingali, Kartik Anand

**Affiliations:** 1 Houston Methodist Cancer Center, Houston Methodist Hospital, 6445 Main Street, Outpatient Center, 24th Floor, Houston, TX 77030, USA; CGentilleSanchez@houstonmethodist.org (C.G.); spingali@houstonmethodist.org (S.R.P.); 2 Houston Methodist DeBakey Heart and Vascular Center, 6565 Fannin St, Houston, TX 77030, USA; BTrachtenberg@houstonmethodist.org; 3 Callahan Cancer Center, Great Plains Health, 601 W Leota St, North Platte, NE 69101, USA

**Keywords:** CAR-T, cardiotoxicity, hypotension, heart failure, cytokine release syndrome, leukemia, lymphoma

## Abstract

Chimeric antigen receptor T-cells (CAR-T) are improving outcomes in pediatric and adult patients with relapsed or refractory B-cell acute lymphoblastic leukemias and subtypes of non-Hodgkin Lymphoma. As this treatment is being increasingly utilized, a better understanding of the unique toxicities associated with this therapy is warranted. While there is growing knowledge on the diagnosis and treatment of cytokine release syndrome (CRS), relatively little is known about the associated cardiac events that occur with CRS that may result in prolonged length of hospital stay, admission to the intensive care unit for pressor support, or cardiac death. This review focuses on the various manifestations of cardiotoxicity, potential risk factors, real world and clinical trial data on prevalence of reported cardiotoxicity events, and treatment recommendations.

## 1. Introduction

**Figure 1:**
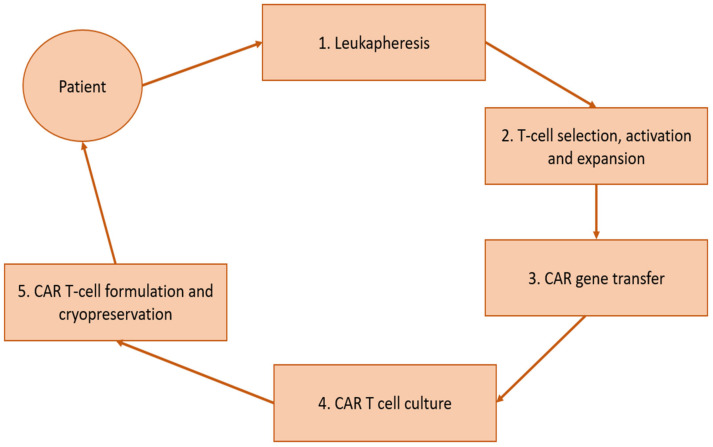
Summary of CAR-T formulation and administration.

Chimeric antigen receptors (CAR) are synthetic immunoreceptors comprised of a single-chain variable fragment acquired from an immunoglobulin with an affinity directed toward a specific tumor antigen, and an intracellular signaling moiety connected by transmembrane domains [1,2,3]. The genetic sequencing is introduced to a patient’s T-lymphocytes ex vivo via lentivirus or non-viral vectors, which are than expanded and returned to the circulation of a patient that has received lymphodepleting chemotherapy, commonly fludarabine and cyclophosphamide [4,5]. Once the modified cells are introduced to the patient’s circulation, the CAR-T cells can preferentially target tumor cells with aberrant expression of a specific antigen (Figure 1) [6].

The development of genetically modified CAR-T cells targeting CD19, an antigen that is frequently over-expressed in various B-cell malignancies, is changing the therapeutic landscape in pediatric and adult patients with relapsed/refractory (r/r) B-cell acute lymphoblastic leukemia (B-ALL) and subtypes of non-Hodgkin Lymphoma (NHL). Following the results of the study of Efficacy and Safety of CTL019 in Adult DLBCL Patients (JULIET trial) and study of Tisagenlecleucel in children and young adults with B-cell lymphoblastic leukemia (ELIANA trial), Tisagenlecleucel (CTL019; Novartis Pharmaceuticals) was approved by the food and drug administration (FDA) for B-ALL in 2017 and large B-cell lymphoma (LBCL) (including diffuse large B-cell lymphoma [DLBCL], high-grade B-cell lymphoma [BCL], and DLBCL arising from follicular lymphoma [FL]) in 2018. These approvals encompass pediatric and young adult patients up to the age of 25 years with second or later disease relapse or that are refractory to two or more lines of systemic therapy [7,8]. Following the results from the safety and efficacy of KTE-C19 in adults with refractory aggressive non-Hodgkin lymphoma (ZUMA-1 trial), Axicabtagene Ciloleucel (KTE-C19; Kite Pharma Incorporated) was approved by the FDA in 2017 in adults with LBCLs (DLBCL, primary mediastinal large B-cell lymphoma [PML], high-grade BCL, and DLBCL arising from FL) that are relapsed or refractory to two or more lines of systemic therapy [9]. In 2020, following the study on KTE-X19 CAR T cell therapy in relapsed or refractory mantle-cell lymphoma (ZUMA-2 trial), Brexucabtagene Autoleucel (KTE-X19, Kite Pharma Incorporated) was approved by the FDA for the treatment of adult patients with r/r mantle cell lymphoma (MCL) [10].

While these agents are reporting unparalleled response rates ranging from 50–93% in the r/r setting [7,8,9,10], these therapies carry unique and potentially significant toxicities. In clinical trials and retrospective institutional studies, cytokine release syndrome (CRS) is a frequently encountered toxicity. While there is a growing body of literature aimed at studying and ameliorating the potential impacts of these toxicities following CAR-T administration, there is little data on cardiotoxic events. While uncommonly reported in the pivotal trials [11], as the treatment population receiving CD19 CAR-T grows, understanding potential cardiac events that may occur, predisposing risk factors, and management recommendations is necessary to improve non-relapse related outcomes. The following review aims to provide a comprehensive review on cardiac toxicities, as well as diagnostic and treatment considerations for pediatric and adult patients undergoing CD19 CAR-T infusions.

## 2. Cardiotoxicity

Cardiac events following CD19 CAR-T infusion frequently occur as a complication arising in patients with grade 3–4 CRS (Table 1) [12,13,14,15]. Upon recognition of the tumor antigen, CAR-T cells release pro-inflammatory cytokines including interferon gamma (IFNγ), interleukin (IL)-1, IL-2 receptor alpha (RA), tumor necrosis factor alpha (TNFα), and IL-6 to induce a cytotoxic response [15]. The release of these cytokines, particularly IL-6, also plays a role in the pathogenesis of CRS, including the recruitment of additional T-lymphocytes [16,17]. High levels of circulating IL-6 can also lead to myocardial stunning which may be clinically indistinguishable from septic cardiomyopathy [18,19]. Furthermore, CRS results in the activation of prostaglandins, which can also impart a risk of cardiotoxic events, such as tachycardia and hypotension [15,16]. It is also possible that CAR-T results in a direct or “off-target” cardiotoxic injury as a result of cross-reactivity between T-cells and Titin, a reaction observed in an early CAR-T targeting melanoma-associated antigen 3 (MAGEA3) [15,18,20].

A wide array of cardiac events has been reported in both pediatric and adult patients, including sinus tachycardia, arrhythmias, ST segment changes on the electrocardiogram (ECG), left ventricular systolic dysfunction, decompensated heart failure, life-threatening hypotension requiring vasopressor support, and rarely, cardiac death (Table 2). In the pivotal trials, patients with pre-existing or recent cardiovascular events were excluded from enrollment (Table 3), but many patients included in these trials had previously received therapies that increase the risk of cardiac disease, including treatment with anthracycline containing treatment regimens, irradiation, and allogenic stem cell transplantation. Outside of the clinical trials, data reporting on cardiotoxic events are starting to accumulate. Currently, cardiac disease is not an absolute contraindication to proceeding with CAR-T [21,22,23].

### 2.1. Cardiotoxicity in Pediatric and Young Adult Patients

Hypotension is frequently reported in pediatric patients and is often a complication of CRS. In the ELIANA trial, 29% of patients developed hypotension, and 17% were grade 3–4 (Table 1 and Table 4) [8]. Other studies of pediatric and young adult patients have found that 18–38% of patients will develop grade 3–4 hypotension (Table 5) [8,24,25,26,27], with 13–27% of these patients requiring vasopressor support [24,25,28,29], and up to one-third of patients requiring admission to the intensive care unit (ICU) [25]. In a retrospective study by Fitzgerald et al., 13 patients that developed cardiovascular toxicity developed fluid-refractory shock that necessitated the use of alpha agonists. Ten of these patients required additional vasoactive substances. In this cohort, cardiovascular dysfunction developed at a median of 5 days after the infusion and lasted a median of 4 days. All patients had a preceding fever prior to the onset of shock [30].

A wide array of other cardiac events is reported in the pediatric and young adult population, frequently resulting as a sequela of hypotension and CRS (Table 5). In the ELIANA trial, 3 (4%) patients developed left ventricular dysfunction, 2 (2.7%) patients developed cardiac failure, and 3 (4%) had cardiac arrest (Table 4) [8]. In a retrospective study by Burstein et al., 10 (41%) of the 24 patients that developed hypotension requiring inotropic support went on to develop systolic dysfunction, and one patient had cardiac arrest. Six of these patients required milrinone, and 5 of these patients required additional inotropic support including dopamine, epinephrine, norepinephrine, or vasopressin. Six of these patients had ST segment changes. All patients had grade 3–4 CRS. [29]. In a retrospective study of pediatric patients that developed cardiotoxicity by Shalabi et al., 6 (16%) patients with CRS developed cardiac dysfunction, and all required monitoring in the intensive care unit. No cardiotoxicity was reported in the patients that did not develop CRS. Patients with cardiac dysfunction in this study were more likely to have earlier CRS, more severe CRS, receive tocilizumab, and have a lower baseline global longitudinal strain (GLS). In this patient cohort, of the 37 patients that developed CRS, 9 (24%) developed hypotension requiring vasopressors, with 3 of these patients requiring two or more vasopressor agents [28].

In children, cardiotoxic complications associated with CAR-T appear to largely be self-limited, with most patients recovering cardiac function back to pre-treatment baseline, including patients that had cardiac arrest [28,29]. In the study by Shalabi et al, all 6 individuals with heart failure had recovery by 3 months, and in the retrospective study by Burstein, 6 of the 7 with impaired systolic function had recovery by 6 months of follow up. None of the cardiac events contributed to mortality [28,29].

### 2.2. Cardiotoxicity in Adult Patients

In the JULIET, ZUMA-1, and ZUMA-2 trials assessing CD19-CAR-T cells in adults, hypotension ranged from 26–59%, and hypotension or shock requiring pressor support occurred in 9–22% of patients. Tachycardia was also reported between 11–39% of patients, but cardiac arrest and heart failure were not reported (Table 4) [7,9,10]. Outside of the pivotal trials, the first retrospective study assessing cardiovascular outcomes came from Alvi et al. [31]. In this analysis involving patients treated with Axicabtagene Ciloleucel, Tisagenlecleucel, and investigational CD19 CAR-T, 17 (12%) developed cardiovascular events with a median time of 21 days to the event, and a median follow up of 10 months. There were 6 cardiac deaths, 6 accounts of acute heart failure, and 5 occurrences of new-onset arrhythmias (Table 6). There appeared to be a close relationship between cardiac events, cardiac injury, and CRS; all patients had > grade 2 CRS, and 16 patients with cardiotoxicity had a positive troponin out of a total of 29 patients in the entire cohort with a positive troponin [31].

In a recent study assessing major cardiac events (MACE) including cardiovascular death, symptomatic heart failure, acute coronary syndrome, ischemic stroke, and de novo cardiac arrhythmia in patients that received CAR-T, Lefebvre found that 31 (21.4%) patients developed 41 cardiac events within a median of 11 days, and over a follow-up period of 753 days. Kaplan–Meier methodology found that the cumulative incidence for a major cardiac event was 17% at 30 days, 19% at 6 months, and 21% at 12 months following CD19-CAR-T infusion, suggestive of a longer-term risk of cardiovascular events [32]. In another study conducted by investigators at the Dana Farber Cancer institute, the incidence of cardiomyopathy was assessed in 187 patients receiving CD19 CAR-T for NHL. In this study 155 (83%) patients developed ≥2 CRS, and there were 116 with serial echocardiograms available for evaluation. Of these 116, 12 (10.3%) developed new (11) or progressive (1) cardiomyopathy within a median of 12.5 (2–24) days following CAR-T infusion. Of these, 11 had ≥2 CRS, and all were treated with Tocilizumab. In addition, 5 (42%) required vasopressor support. Of these 12, the ejection fraction improved in 9 of the 12 patients over 168.5 (3–471) days, with normalization 6 and partial recovery in 3. The 3 without recovery in cardiac function died [18].

Contrary to what is seen in the pediatric and young adult patients that develop cardiotoxicity, adult patients receiving CD19 CAR-T agents that develop cardiotoxicity do not always have resolution of their cardiac events, and in some cases, have fatal events. While there are risk factors becoming recognized for patients that may be at risk of developing CRS and association with cardiotoxicity, there needs to be a standardized pre-treatment protocol for screening these patients with recommendations on how to proceed for patients with increasingly recognized risks for cardiotoxicity.

## 3. Risks for Developing Cardiotoxic Events

Cardiotoxicity is frequently a sequela of CRS, so understanding risks that may predispose to CRS should be considered in patients, especially those with pre-existing cardiovascular disease. Patients that are at risk of developing CRS are patients with high disease burden, an older age at the time of infusion, higher-intensity lymphodepleting regimen, utilization of fludarabine and cyclophosphamide during lymphodepleting chemotherapy, higher infused CAR T-cell doses, use of unselected bulk CD8^+^ T cells, high levels of CTL019^+^ CD8 and CD3 cells, the presence of inflammatory markers including a higher peak of C-reactive protein, and severe thrombocytopenia [25,27,33,34] (Table 7).

Knowledge or risk factors directly contributing to cardiotoxicity are largely limited to retrospective studies. In pediatric and young adult patients, the risk of developing hypotension requiring inotropic support appears to be associated with a pre-treatment blast percentage >25% on bone marrow biopsy, lower baseline ejection fraction or GLS, or pre-existing diastolic dysfunction [28,29]. Studies have not found previous anthracycline-based chemotherapy, radiation exposure, or history of stem cell transplantation to be associated with the risk of developing clinically significant hypotension or cardiac dysfunction [16,29] (Table 7).

In adults, Alvi et al. reported that concomitant CRS, troponin elevation, and the time of onset of elevated troponin in CRS to the first administration of tocilizumab, are associated with an increased risk of developing a cardiovascular event [31]. Lefebvre et al. found that patients at risk for developing MACE were older, had a higher prevalence of baseline cardiovascular risk factors at baseline, higher baseline creatinine, and grade 3–4 CRS [32]. In addition, prior, aspirin use, statin use, and insulin use had a higher association with MACE [32]. The subtype of malignancy did not seem to impart a risk of cardiotoxicity [32]. Ganatra et al. found that adults that were older, had high-grade CRS, hyperlipidemia, known coronary artery disease, or use of renin-angiotensin inhibitors and beta-blockers at baseline had a higher risk of developing post-CAR-T cardiomyopathy [18] (Table 7).

## 4. Management and Treatment

### 4.1. Pre-CAR-T Infusion Cardiovascular Considerations

**Figure 2:**
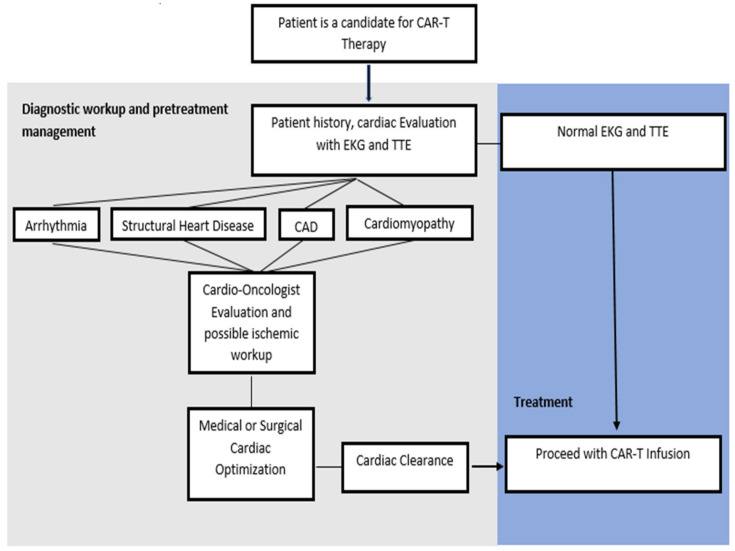
Pre-operative assessment. CAR-T: Chimeric antigen receptor T-cells; EKG: Electrocardiogram; TTE: Transthoracic echocardiogram; CAD: Coronary artery disease.

Patients and pertinent risk factors that may predispose to cardiotoxicity should be identified prior to initiation of CAR-T infusion. In retrospective studies, the timing from CAR-T infusion to reported cardiac event ranged from a median of 5–21 days [18,28,29,30,31,32]. While baseline cardiac evaluation is typically institution dependent, the American Council of Cardio-Oncology recommends the consideration of a baseline ECG and echocardiographic assessment of cardiac function. Specifically, the presence of baseline arrhythmias, structural heart disease, or coronary artery disease (CAD) should be assessed [35]. If a patient is found to have systolic or diastolic dysfunction, valvular disease, cardiomyopathy, CAD, or pre-existing risk factors, then they should be referred for evaluation by a Cardio-Oncologist for further risk stratification and optimization of cardiovascular function [35,36]. Ischemic burden should be assessed with stress test imaging in patients with known CAD or risk factors that may predispose to cardiovascular decompensation [35,36]. Prior to CAR-T infusion, patients with pre-existing systolic and diastolic dysfunction should be volume optimized and monitored closely during infusion due to the risk for fluid shifts [35]. In addition, outpatient medications should be reviewed, particularly antihypertensives, antiplatelets, and other anticoagulants given the risk of hemodynamic compromise after CAR-T infusion as well as cytopenias due to lymphodepletion (Figure 2) [35,37].

### 4.2. Clinical Monitoring during and after CAR-T Infusion

**Figure 3:**
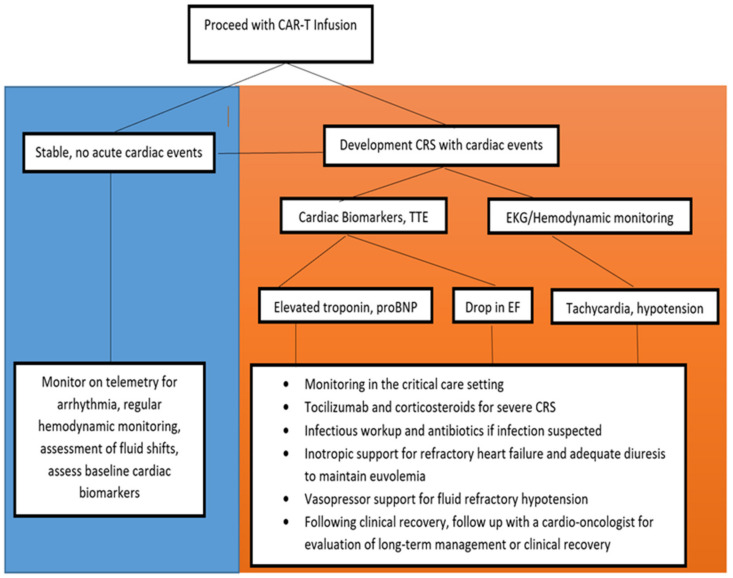
Management consideration in patients that develop CRS that may be at risk for cardiotoxicity. CRS: Cytokine release syndrome. TTE: Transthoracic echocardiogram; EKG: Electrocardiogram; EF: Ejection fraction; proBNP: Brain natriuretic peptide.

Patients that develop ≥grade 2 CRS are at a higher risk of developing cardiotoxicity, so close clinical monitoring for the development of CRS is crucial. Although recommendations vary, monitoring may include blood pressure checks, 12-lead ECGs, telemetry, and cardiac biomarker assessments including cardiac troponin and brain natriuretic peptide in patients that are showing clinical evidence of CRS [35,36,38]. If hypotension or tachycardia develops, patients should undergo volume resuscitation. However, close attention should be paid to symptoms of vascular leak and pulmonary edema [37]. If refractory to fluid boluses, transfer to the ICU for close hemodynamic monitoring and vasopressor initiation should be considered. Reassessment of left ventricular function with an echocardiogram can help elucidate a cardiogenic component to shock [35] (Figure 3).

Although troponemia has been associated with an increased risk of developing cardiovascular events after CAR-T infusions [31], there is limited data on the benefit of obtaining routine biomarker assessments. While additional studies need to be done in both pediatric and adult patients to corroborate this, it should be considered particularly in those that have known pre-existing cardiovascular disease.

### 4.3. Management of Cardiovascular Events

There are currently no formally accepted guidelines for the management of CAR-T induced cardiotoxicity. Data for specific cardiovascular interventions are lacking; however, treatment recommendations largely revolves around supportive care, including hemodynamic support and management of the precipitating CRS (Figure 3).

#### 4.3.1. Supportive Care

Supportive care must be initiated in patient with hypotension, myocardial depression, arrhythmias, and sinus tachycardia. In hypotension, initial treatment with intravenous crystalloids is recommended [35]. Close clinical monitoring to diagnose refractory hypotension and acute fluid shifts predisposing to capillary leak and respiratory failure is important [37]. Patients with refractory hypotension should be initiated on vasopressor support and monitored closely in the ICU. If there is associated tachycardia and fever, appropriate infectious workup and broad-spectrum antimicrobial agents should be initiated [39]. In the setting of depressed myocardial dysfunction or cardiogenic shock, inotropic support with agents like dobutamine can also be considered [36].

#### 4.3.2. IL-6 Inhibitor Therapy

CRS is a systemic inflammatory response mediated by the release of cytokines such as IL-6, IL-10, interferon gamma and tumor necrosis factor alpha. Once secreted, IL-6 has pro-inflammatory effects which play a substantial role in the pathogenesis of CRS including capillary leak, hypotension, complement activation and myocardial dysfunction [40,41]. Tocilizumab is a monoclonal antibody that inhibits the binding of IL-6 to its receptor. It acts on both membrane bound and soluble IL-6 receptors thereby inhibiting downstream IL-6 signaling [40]. It was approved by the FDA for use in severe or life-threatening CAR-T mediated CRS in adult and pediatric patients older than 2 years alone or in combination with steroids, following the results of a retrospective analysis of pooled data from 5 clinical trials [42].

The management of CRS has important implications in cardiotoxicity events. Alvi et al. reported that in patients with troponemia following CAR-T infusion, the risk of a cardiovascular event increases with each 12-h delay in administration of tocilizumab [31]. It is generally accepted to start tocilizumab in the presence of hemodynamic instability requiring vasopressor support, increasing oxygen requirement and/or evidence of end-organ dysfunction (unstable arrhythmia, myocardial infarction, cardiomyopathy) [35,37,43].

An alternative IL-6 inhibitor, siltuximab, has also been used in CRS. Its mechanism differs to that of tocilizumab in that is directly binds to circulating IL-6 [37,40]. Prior studies have administered it either interchangeably with tocilizumab or as an option in refractory CRS [38]. At this time, it is not FDA approved and its use is investigational, however, recommendations are to consider it in patients with CRS that have not responded to tocilizumab and/or corticosteroids [35,41,44]. It is not known if expedient administration of siltuximab would similarly reduce the risk of developing cardiac events in patients with troponemia, like what is seen with tocilizumab [31].

#### 4.3.3. Corticosteroid Therapy

The use of corticosteroids has proven to be effective in CRS, particularly when it is severe and refractory to other interventions [36,44]. Although practices vary, it is commonly considered a 2nd line agent. However, it may also be administered in conjunction with tocilizumab in cases of severe CRS [37,38,40].

Further research is needed to define the role and safety of corticosteroids for treatment of cardiotoxicity events related to, or independent of CRS. “Stress dose” corticosteroids are frequently used in cases of refractory hypotension stemming from septic shock, and in other cases in which the hypothalamic-pituitary access (HPA) may be significantly impaired [45,46]. However, in the setting of immune activation, pro-inflammatory cytokines such as IL-6 are known to augment the HPA axis and increase the level of circulating corticosteroids [47]. While administration of systemic corticosteroids does not impart a detrimental impact on CAR-T efficacy, its use to treat or prevent cardiac events is poorly understood. It is becoming routinely used in CRS, but in a study of pediatric patients, administration of steroids did not reduce the risk of developing cardiac dysfunction (*p* = 0.11) [28]. Additional data related to corticosteroid use and cardiotoxicity remain sparse, and additional studies are needed to better understand the therapeutic applications.

#### 4.3.4. The Role of IL-1 Therapy

There are ongoing studies exploring the use of other agents in CRS management. IL-1 is a particularly enticing target given its pro-inflammatory function, pathophysiologic role in CRS, and its role in cardiovascular disease [48]. Phase II and III trials have shown that blocking IL-1 may prevent recurrent atherothrombotic cardiovascular events, increase exercise capacity in heart failure patients and prevent heart failure following a myocardial infarction [48]. Anakinra is an IL-1 inhibitor that is FDA approved for rheumatoid arthritis. However, pre-clinical data have indicated a potential role in CRS [40,41]. Two animal model studies have reported that IL-1 blockade can prevent CRS, thereby reducing mortality [49,50]. A case report of two patients using tocilizumab and anakinra (200 mg subcutaneous three times daily) in a patient treated with anti-BCMA CAR T was published recently [51]. Only one dose of tocilizumab was given and a taper of anakinra over the following 10 days was done. The authors noticed significant improvement of the patient’s symptoms shortly after anakinra was initiated. They theorize that IL-1 blockage decreased the duration and severity of the CRS as well as the need for further tocilizumab doses [51]. In addition, a phase II clinical trial to explore the role of anakinra further is underway (NCT04359784).

#### 4.3.5. The Role of TNF-α Therapy

TNF-α is a pro-inflammatory cytokine that is elevated following administration of CD19 CAR-T therapy [52]. Therefore, it is possible that subsets of patients with CRS may benefit from anti-TNFα monoclonal antibodies infliximab or soluble TNFα receptor, etanercept. There have been rare reports of the utilization of TNF-α inhibitor therapy for CRS arising from CAR-T therapy. Lee et al. reported on a case of grade 3 CRS arising in a 19-year-old female being treated with CAR-T for EBV-associated lymphoma [13]. Within hours of receiving treatment with etanercept, methylprednisolone, dopamine, and norepinephrine, her symptoms resolved [13]. Furthermore, in a small study of 8 patients with r/r multiple myeloma treated with LCAR-B38M (anti-BCMA CAR-T cells), 3 patients were treated with etanercept (either 25 mg or 50 mg) with excellent clinical response [53]. As the number of clinical trials for CAR-T in MM increase, the role of TNF—α may need to be further explored for severe CRS [54].

Neither of the reports above involved treatment with CD19 CAR-T cells. Whether these patients may derive similar benefit is not currently known. In a report of 2 pediatric patients with pre-B-cell ALL, 1 patient developed severe CRS, which responded to treatment consisting of etanercept, tocilizumab, and corticosteroids [55]. Additional studies are needed to determine if patients receiving CD19 CAR-T cells that develop grade 3 or 4 CRS should be considered to receive anti- TNF-α therapy.

## 5. Conclusions

As the therapeutic applications of CD19 CAR-T continues to grow in the relapsed/refractory setting of B-ALL and NHL, recognition, and management of associated toxicities in the standard of care setting is crucial. While the unique toxicities associated with CD19 CAR-T such as CRS is well-studied, there remains limited data on identification and management of cardiac toxicities. While this review highlights the currently available literature on risk factors and treatment considerations of cardiotoxicitiy in the pediatric and adult populations, there remains a paucity of guidelines available. Future studies are needed to better direct the management of these patients to reduce the morbidity following CAR-T administration.

## Figures and Tables

**Table 1:** Grading Criterias for CRS in the pivotal and included retrospective studies.

Penn Criteria [12]				
	Grade 1	Grade 2	Grade 3	Grade 4
	Mild reaction: Treated with supportive care, such as antipyretics, antiemetics	Moderate reaction: Some signs of organ dysfunction (grade 2 creatinine or grade 3 LFTs) related to CRS and not attributable to any other condition. Hospitalization for management of CRS-related symptoms, including neutropenic fever and need for i.v. therapies (not including fluid resuscitation for hypotension)	More severe reaction: Hospitalization required for management of symptoms related to organ dysfunction, including grade 4 LFTs or grade 3 creatinine, related to CRS and not attributable to any other condition. Hypotension treated with multiple fluid boluses or low-dose vasopressors. Coagulopathy requiring fresh frozen plasma, cryoprecipitate, or fibrinogen concentrate. Hypoxia requiring supplemental oxygen (nasal cannula oxygen, high-flow oxygen, CPAP, or BiPAP)	Life-threatening complications such as hypotension requiring high-dose vasopressors. Hypoxia requiring mechanical ventilation
Lee Criteria [13]				
	Grade 1	Grade 2	Grade 3	Grade 4
	Symptoms are not life-threatening and require symptomatic treatment only (fever, nausea, fatigue, headache, myalgias, malaise)	Symptoms require and respond to moderate intervention: Oxygen requirement < 40% FiO_2_, OR hypotension responsive to i.v. fluids or low dose of one vasopressor, OR grade 2 organ toxicity *	Symptoms require and respond to aggressive intervention: Oxygen requirement ≥ 40% FiO_2_, OR Hypotension requiring high-dose or multiple vasopressors, OR grade 3 organ toxicity, or grade 4 transaminitis	Life-threatening symptoms: Requirement for ventilator support, OR grade 4 organ toxicity (excluding transaminitis)
ASTCT Consensus Criteria [14]				
	Grade 1	Grade 2	Grade 3	Grade 4
	Temperature ≥ 38 °C, no hypotension, no hypoxia	Temperature ≥ 38 °C, with hypotension not requiring vasopressors, and/or hypoxia requiring low flow nasal cannula	Temperature ≥ 38 °C, with hypotension requiring vasopressors with or without vasopressin, and/or hypoxia requiring high-flow nasal cannula, facemask, nonrebreather mask, or venturi mask	Temperature ≥ 38 °C, with hypotension requiring multiple vasopressors (excluding vasopressin), and/or hypoxia requiring positive pressure (CPAP, BiPAP, intubation, and mechanical ventilation)

* Cardiac (tachycardia, arrhythmias, heart block, or decrease in ejection fraction), respiratory (tachypnea, pleural effusion, or pulmonary edema), renal (acute kidney injury, increase in serume creatinine level, or decrease in urine output), gastrointestinal (nausea, vomiting, or diarrhea), hepatic (increase in serum alanine aminotransferase, aspartate aminotransferase, or bilirubin level), coagulopathy (disseminated intravascular coagulation), or dermatologic (rash).

**Table 2:** Summary of reported cardiovascular events associated with CD19-CAR-T.

Reported Cardiotoxic Events with FDA Approved CD19 CAR-T
Tachycardia
Hypotension
Fluid refractory hypotension
Pulmonary Edema
Depressed left ventricular function
Cardiac failure
Cardiac failure requiring inotropic support
Elevated troponin
Arrhythmia
ST-segment changes
Cardiac arrest

**Table 3:** Cardiac exclusion criteria for pivotal trials of CD19-CAR-T. ECG: Electrocardiogram.

CD19-CAR-T Infusion	Tisagenlecleucel	Tisagenlecleucel	Axicabtagene Ciloleucel	Brexucabtagene Autoleucel
Trial	ELIANA [8]	JULIET [7]	ZUMA-1 [9]	ZUMA-2 [10]
Pertinent cardiovascular trial exclusion criteria	-Left Ventricular systolic function ≤ 28% confirmed by echocardiogram -Left ventricular ejection fraction ≤ 45% confirmed by echocardiogram or multigated acquisition images within 7 days of screening	-Unstable Angina or MI within 6 months of planned infusion -Uncontrolled arrhythmia	-EF < 50% determined by transthoracic echocardiogram -Evidence of pericardial effusion -Presence of clinically significant ECG findings	-Cardiac ejection fraction < 50% -Evidence of pericardial effusion -Clinically significant electrocardiogram findings -Myocardial infarction, cardiac angioplasty or stenting, unstable angina, active arrhythmias, or other clinically significant cardiac disease within 12 months of enrollment -Cardiac atrial or cardiac ventricular lymphoma involvement

**Table 4:** Summary of outcomes and reported cardiotoxic events in the pivotal phase 2 trials leading to FDA approval of Tisgenlecleucel, Axicabtagene Ciloleucel, and Brexucabtagene Ciloleucel.

FDA Approved CD19-CAR-T	Tisagenlecleucel	Tisagenlecleucel	Axicabtagene Ciloleucel	Brexucabtagene Autoleucel
Trial	JULIET [7]	ELIANA [8]	ZUMA-1 [9]	ZUMA-2 [10]
Disease	Adult LBCL	Pediatric B-ALL	Adult LBCL	Adult MCL
Study Phase	2	1–2	2	2
Patients Studied in Efficacy Analysis	93	75	101	68
Objective Response Rate	50%	83%	82%	93%
Complete Response	40%	60%	54%	67%
12 month RFS/PFS	65%	59%	44%	61%
12 month OS	49% (estimated)	76%	59%	83%
Patients Studied in Safety Analysis	111	75	101	68
Percent with any Grade AE	100%	100%	100%	100%
CRS	64 (58%)	58 (77%)	94 (93%)	61 (91%)
CRS Grading System	Penn Criteria [12]	Penn Criteria [12]	Lee Criteria [13]	Lee Criteria [13]
Tocilizumab Use	16 (14%)	36 (48%)	49 (48.5%)	42 (61.8%)
Hypotension	29 (26%)	22(29%)	60 (59%)	35 (51%)
Hypotension requiring inotropic support or shock	8 (9%)	13 (17%)	14 (14%)	15 (22%)
Pulmonary Edema	NR	5 (6.7%)	NR	NR
Left Ventricular Dysfunction	NR	3 (4.0%)	NR	NR
Cardiac Arrest	NR	3(4.0%)	NR	NR
Cardiac Failure	NR	2 (2.7%)	NR	NR
Tachycardia	12 (11%)	3 (4.0%)	39 (39%)	21 (31%)

B-ALL: B-cell Acute Lymphoblastic Leukemia; RFS: Relapse Free Survival; PFS: Progression Free Survival; OS: Overall Survival; AE: Adverse Events; CRS: Cytokine Release syndrome; LBCL: Large B-cell Lymphoma; MCL: Mantle Cell Lymphoma.

**Table 5:** Summary of cardiotoxicity in retrospective pediatric assessments.

CD19-CAR-T Cardiovascular Events	Shalabi et al. (2020) [28]	Burstein et al. (2018) [29]	Fitzgerald et al. (2017) [30]
Patient Population	Pediatric (*n* = 52)	Pediatric (*n* = 98)	Pediatric (*n* = 39)
Treatment Indication			
B-ALL	50 (96.1%)	90 (97%)	39 (100%)
NHL	2 (3.9%)	1 (1%)	0
Multiple Myeloma	0	0	0
T-ALL	0	1 (1%)	0
PML	0	1 (1%)	0
CRS Grading System	Penn Criteria [12] ASTCT Consensus Criteria [14]	Penn Criteria [12]	Penn Criteria [12]
Cardiotoxic Events			
Pre-existing Cardiomyopathy/Structural Disease/Arrhythmia	6 (11.5%)	10 (11%)/1(5%)	NR
Hypotension Requiring Inotropic Support	9 (24.3%)	24 (24%)	13 (33%)
Troponemia	NR	NR	NR
Ventricular Systolic Dysfunction	6 (11.5%)	10 (10%)	1 (2%)
Tachycardia	36 (69.2%)	NR	NR *
Arrhythmia	NR	NR	NR
ST segment changes	NR	6 (6%)	NR
Cardiac Arrest/ Cardiac Death	1 (2.7%)	0	NR
Required Tocilizumab	14 (37.8%)	21 (21%)	13 (33%)

* Number with tachycardia not reported. B-ALL: B-cell Acute Lymphoblastic Leukemia; NHL: non-Hodgkin Lymphoma; T-ALL: T-cell Acute Lymphoblastic Leukemia; PML: Primary Mediastinal Large B-cell Lymphoma.

**Table 6:** Summary of cardiotoxicity in retrospective adult assessments.

CD19-CAR-T Cardiovascular Events	Ganatra et al. (2020) [18]	Alvi et al. (2019) [31]	Lefebvre et al. (2020) [32]
Patient Population	Adults (*n* = 187)	Adult (*n* = 137)	Adult (*n* = 145)
Treatment Indication			
B-ALL	1 (0.5%)	0	36 (25%)
NHL	185 (98.7%)	119 (88%)	43 (30%)
Multiple Myeloma	0	11 (8%)	0
T-ALL	0	0	0
PML	1 (0.5%)	0	0
CLL	0	0	66 (46%)
CRS Grading System	Lee Criteria [13]	Lee Criteria [13]	ASTCT consensus Criteria [14]
Number with Cardiotoxic event	12 (6.4%)	17 (12%)	31 (21.3%)
Pre-existing Cardiomyopathy/Structural Disease/Arrhythmia	1 (0.5%)/4 (2.1%)/3 (1.6%)	5 (3.6%)/10 (7.3%)/18 (13%)	1 (0.7%)/5 (3.4%)/5 (3.4%)
Hypotension/shock Requiring Inotropic Support	5 (2.6%)	6 (4%)	33 (22.7%)
Troponemia	NR	29 (21%)	NR
CHF/Ventricular Systolic Dysfunction	12 (6.4%)	8 (6%)	21 (14.5%)
Sinus Tachycardia	NR	6 (4.4%)	NR
Arrhythmia		5 (3.6%)	13 (8.9%)
ST segment changes	NR	NR	NR
Cardiac Arrest/ Cardiac Death	3 (1.6%)	6 (4.4%)	2 (1.4%)
Required Tocilizumab	12 (6.4%)	56 (40.9%)	15 (10.3%)

B-ALL: B-cell Acute Lymphoblastic Leukemia; NHL: non-Hodgkin Lymphoma; T-ALL: T-cell Acute Lymphoblastic Leukemia; PML: Primary Mediastinal Large B-cell Lymphoma. CLL: Chronic Lymphocytic Leukemia; CHF: Congestive Heart Failure.

**Table 7:** Baseline factors that may increase the risk of Cardiotoxicity.

Predictive Risk Factors for CRS [25,27,34,35]	Risk Factors for Cardiotoxicity in Pediatric Patients [28,29]	Risk Factors for Cardiotoxicity in Adult Patients [18,31,32,35]
High disease burden	Pre-Treatment Blasts >25% on bone marrow biopsy	Concomitant CRS (grade 3 or 4 CRS)
High CAR-T dose	Lower Pre-CAR-T Treatment baseline EF	Troponin elevation
High intensity lymphodepleting regimen	Pre-existing diastolic dysfunction	Older Age
Pre-existing endothelial activation		Higher Baseline Creatinine
Severe thrombocytopenia		Aspirin, statin, insulin, beta blocker, RAA medication use
Addition of fludarabine to cyclophosphamide during lymphodepletion		Hyperlipidemia
Higher peak of C reactive protein		CAD
Older patient age		Aortic Stenosis

EF: Ejection Fraction; CRS: Cytokine Release Syndrome; CAD: Coronary Artery Disease; RAA: Renin-angiotensin-aldosterone.

## Data Availability

Not applicable.

## Abbreviations

CAR-T: Chimeric antigen receptor T-cell; CRS: Cytokine release syndrome; CAR: Chimeric antigen receptor; r/r: Relapsed/refractory; B-ALL: B-cell acute lymphoblastic leukemia; NHL: non-Hodgkin lymphoma; DLBCL: Diffuse large B-cell lymphoma; BCL: High-grade B-cell lymphoma; LBCL: Large B-cell lymphoma; FL: Follicular lymphoma; PML: Primary mediastinal large B-cell lymphoma; MCL: Mantle cell lymphoma; IFNγ: Interferon-gamma; IL-1: Interleukin-1; IL-2RA; Interleukin-2 receptor alpha; TNFα: Tumor necrosis factor-alpha; IL-6: Interleukin-6; MAGEA3: melanoma-associated antigen 3; ECG: Electrocardiogram; LFT: Liver function tests; MI: Myocardial infarction; PFS: Progression free survival; RFS: Relapse free survival; OS: Overall survival; AE: Adverse events; T-ALL: T-cell acute lymphoblastic leukemia; MACE: Major cardiac event; CLL: Chronic lymphocytic leukemia; GLS: global longitudinal strain; CAD: Coronary artery disease; RAA: Renin-angiotensin-aldosterone; ICU: Intensive care unit; HPY: Hypothalamic-pituitary axis.
